# Wrong Tissue at the Wrong Place: A Rare Case of Hypopituitarism Secondary to Metastatic Renal Cell Carcinoma

**DOI:** 10.7759/cureus.64172

**Published:** 2024-07-09

**Authors:** Sreekant Avula, Aditya Chauhan, H. Brent Clark, Hind Alameddine

**Affiliations:** 1 Diabetes, Endocrinology, and Metabolism, University of Minnesota, Minneapolis, USA; 2 Diabetes, Endocrinology, and Metabolism, University of Minnesota School of Medicine, Minneapolis, USA; 3 Pathology, University of Minnesota, Minneapolis, USA

**Keywords:** renal, metastasis, panhypopituitarism, carcinoma, pituitary

## Abstract

Metastasis to the pituitary gland is a very rare occurrence. The most common primary cancer that metastasizes to the pituitary are breast cancer and lung cancer. Most of the pituitary metastases are asymptomatic. The most commonly reported symptoms include anterior pituitary dysfunction, visual field defects, headaches, and diabetes insipidus. Metastasis from renal cell carcinoma (RCC) is very rare. Here, we present the case of a 59-year-old male who presented with vision changes, fatigue, low libido, a low appetite, and excessive thirst. The hormonal evaluation was consistent with panhypopituitarism, and he was started on hydrocortisone, levothyroxine, testosterone, and desmopressin. Brain MRI showed a suprasellar enhancing mass that progressively increased in size. He underwent endoscopic endonasal transplanum and transtuberculum approach for tumor removal. Biopsy of the tumor was reported as metastatic RCC. He was later scheduled for a gamma knife. Metastatic RCC to pituitary is rare, with most being asymptomatic, leading to a delay in diagnosis. Treatment of pituitary metastases is not standardized and should be tailored to patients’ clinical conditions, histology, and the presence of extrapituitary metastases. More prospective studies are needed to formulate guidelines for the management of pituitary metastases.

## Introduction

Pituitary involvement in metastatic disease is very rare, with literature reporting an incidence of 1-4%. The majority of the primary tumors originate from the breast and lung [1-3]. In this subgroup, pituitary metastasis secondary to renal cell carcinoma (RCC) has been reported in fewer than 50 cases [1,4]. In contrast to pituitary metastasis from other primaries, RCC metastasis has been reported to commonly involve the anterior pituitary, with the majority of cases presenting with panhypopituitarism [4,5]. The time from the diagnosis of RCC to pituitary metastasis has a wide range from 0 to 27 years [4]. We present the case of a 60-year-old male who presented with hallucinations, visual field defects, and panhypopituitarism and was found to have RCC metastasis to the pituitary gland after an initial diagnosis of RCC 12 years ago with negative surveillance.

This article was previously presented as a meeting poster at the 2023 ENDO Annual Scientific Meeting on June 16, 2023 [6].

## Case presentation

A 60-year-old male with a past medical history of stage II clear-cell RCC status post-right nephrectomy in 2010 with no adjuvant therapy presented to our hospital in November 2022 with visual and olfactory hallucinations for three days along with worsening bitemporal vision loss. Notably, the patient started noticing right-sided peripheral vision loss in early 2021, for which he did not seek any medical attention until September 2021. His visual symptoms progressed, and he also developed excessive fatigue, loss of libido, loss of appetite, and excess thirst. MRI of the brain was performed in Palestine which was concerning for a 2 cm suprasellar enhancing mass arising from the suprasellar cistern and appeared to be inseparable from the pituitary stalk. A hormonal workup in Palestine revealed panhypopituitarism, for which he was started on levothyroxine, desmopressin, hydrocortisone, and testosterone.

Diagnostic assessment

**Figure 1:**
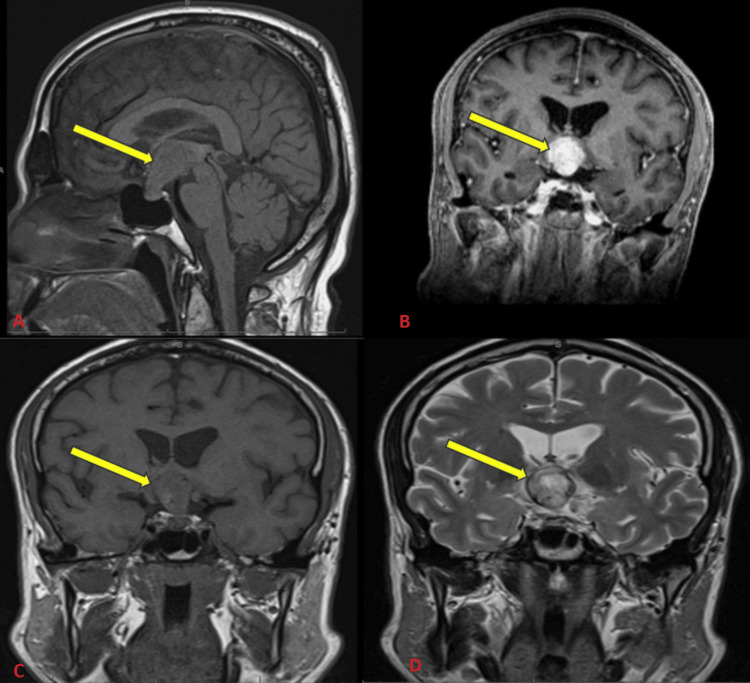
MRI of the brain showing sellar and suprasellar mass measuring 2.1 × 2.3 × 3.8 cm, extending to the suprasellar cistern, and elevating the optic chiasm. A: T1-weighted sagittal view showing hypointense pituitary macroadenoma (yellow arrow). B: T1-weighted coronal view post intravenous contrast diffuse enhancement in the sella region (yellow arrow). C: T1-weighted coronal view showing hypointense macroadenoma (yellow arrow). D: T2-weighted coronal view showing a heterogeneous signal with a rim of hemosiderin and internal heterogeneous iso and hyperintense areas (yellow arrow).

MRI of the brain with and without contrast showed a sellar mass with trace hemorrhage measuring 2.1 × 2.3 × 3.8 cm extending to the suprasellar cistern and edema of the optic tracts and the cerebral peduncle (Figure 1).

The mass had increased in size when compared with MRI in September 2021 (1 × 1.4 × 2 cm). Laboratory investigations showed suppressed follicular-stimulating hormone level (0.5 mIU/mL, 0.5 IU/L; normal range = 1.5-12.5 mIU/mL, 1.5-12.5 IU/L), suppressed luteinizing hormone level (<0.3 mIU/mL, <0.3 IU/L; normal range = 1.7-8.6 mIU/mL, 1.7-8.6 IU/L), mildly elevated prolactin level (54 ng/mL, 54 µg/L; normal range = 4-15 ng/mL, 4-15 µg/L), free T4 level (1.41), thyroid-stimulating hormone level (0.13 µIU/mL, 0.13 mIU/L; normal range = 0.3-4.30 µIU/mL, 0.3-4.30 mIU/L), cortisol level (3.8 µg/dL, 104.88 nmol/L; normal range = 4-22 µg/dL, 110-607 nmol/L), total testosterone (355 ng/dL, 12.31 nmol/L; normal range = 240-950 ng/dL, 8.32-32.93 nmol/L) while on testosterone replacement mid cycle values, insulin-like growth factor-1 level (72 ng/mL, 9.41 nmol/L; normal range = 53-206 ng/mL, 6.92-26.93 nmol/L), and sex hormone-binding globulin levels (3.42 µg/mL, 36 nmol/L; normal range = 1.045-7.6 µg/mL, 11-80 nmol/L) (Table 1).

**Table 1:** Laboratory tests when seen at our clinic.

Lab values	Results	Reference range
Follicular-stimulating hormone	0.5 mIU/ml	1.5–12.5 mIU/mL
Luteinizing hormone	<0.3 mIU/mL	1.7–8.6 mIU/mL
Prolactin	54 ng/mL	4–15 ng/mL
Thyroid-stimulating hormone	0.13 µIU/mL	0.3–4.30 µIU/mL
Free thyroxine	1.41 ng/dL	0.9–1.7 ng/dL
Serum cortisol	3.8 µg/dL	4–22 µg/dL
Total testosterone	355 ng/dL	240–950 ng/dL
Insulin-like growth factor-1	72 ng/mL	53–206 ng/mL
Sex hormone-binding globulin	3.42 µg/mL	1.045–7.6 µg/ml

Treatment

**Figure 2:**
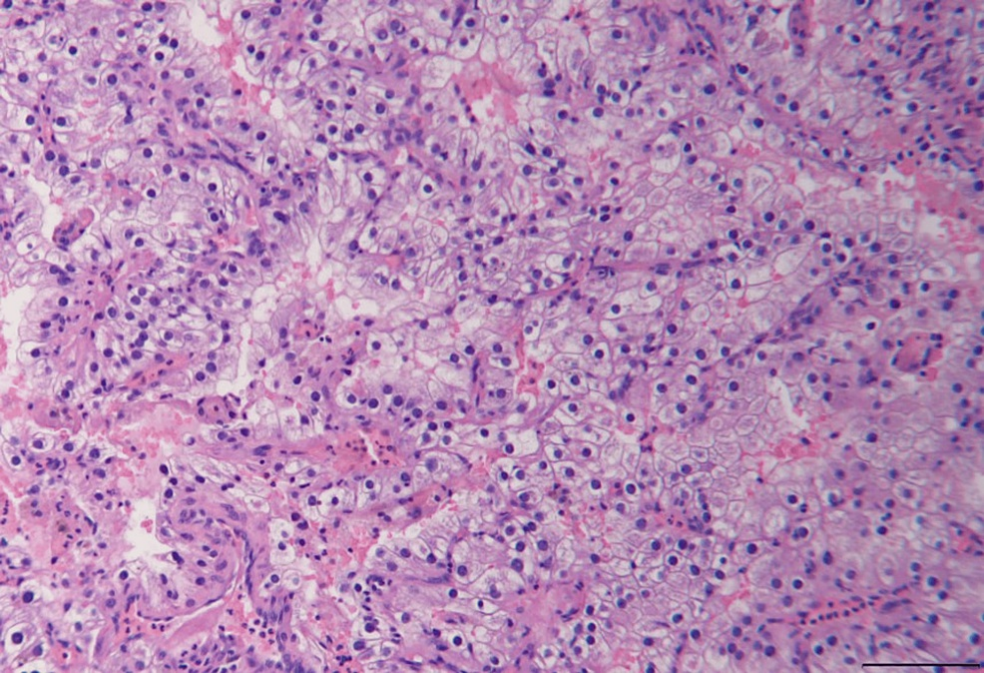
Clear-cell renal carcinoma. Hematoxylin and eosin. Scale bar = 100 µm.

**Figure 3:**
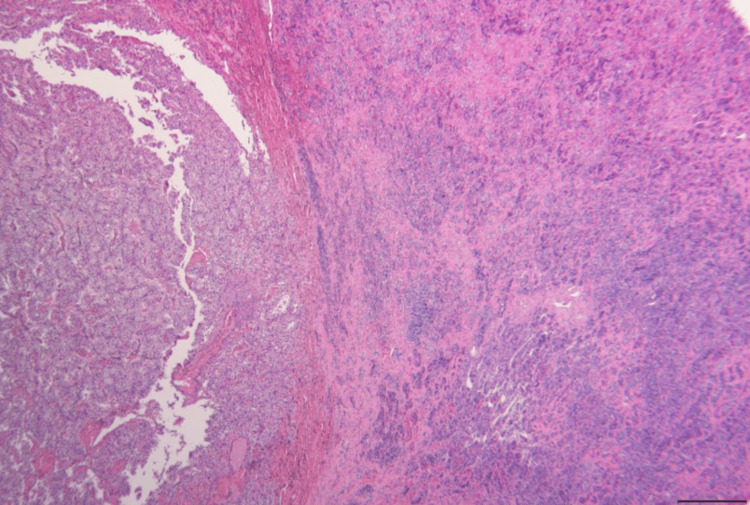
Interface between the metastatic tumor (left) and unaffected pituitary gland (right). Hematoxylin and eosin stain. Scale bar = 500 µm.

**Figure 4:**
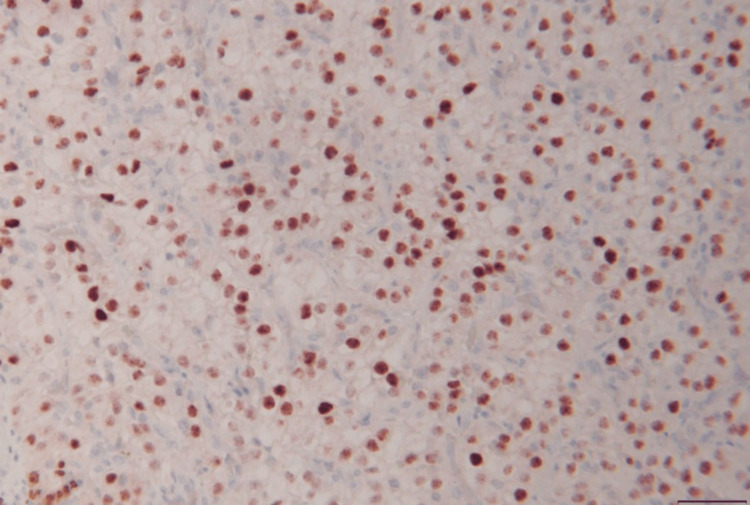
PAX8 immunohistochemical stain with positive nuclear staining. Peroxidase with hematoxylin. Scale bar = 50 µm.

**Figure 5:**
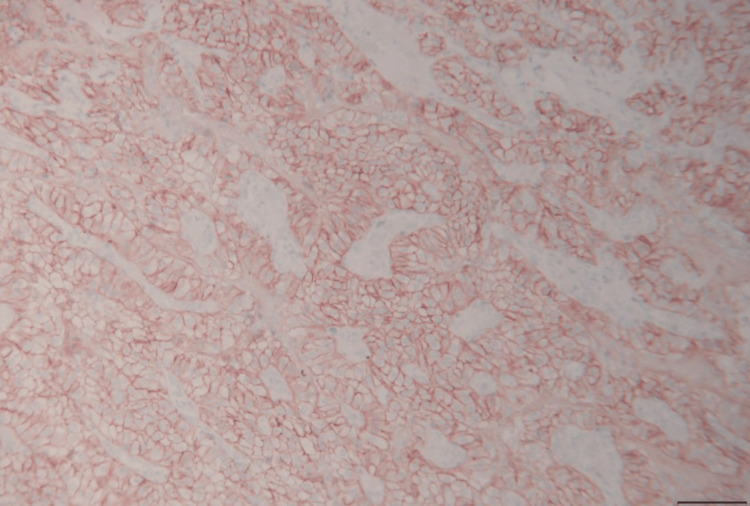
Carbonic anhydrase immunohistochemical stain with membranous staining. Peroxidase with hematoxylin. Scale bar = 50 µm.

The patient underwent resection of the sellar and the suprasellar mass via an endoscopic endonasal approach. The histopathology of the resected mass revealed a discrete neoplasm with the characteristic histology and immunohistochemical profile of metastatic clear-cell renal carcinoma (Figures 2-5).

Postoperatively, he continued levothyroxine 100 µg daily, desmopressin 0.2 mg in the morning and 0.15 at bedtime, and hydrocortisone 10 mg twice daily.

Outcome and follow-up

Postoperatively, CT of the chest, abdomen, and pelvis showed no other suspicious lesions except a prominent right hilar lymph node, although unchanged from previous scans. Considering no other obvious metastatic lesion at that time, the patient received stereotactic body radiation therapy. The patient was recently followed up in the clinic, and he has been doing well on stable doses of levothyroxine, hydrocortisone, desmopressin, and testosterone injections. MRI of the brain from July 2023 showed stable post-surgical changes with no evidence of any suspicious lesion in the brain. The patient continues to follow up with Endocrinology and Oncology.

## Discussion

Metastasis of RCC to the pituitary gland is extremely rare. Pituitary metastasis is reported to be commonly present with diabetes insipidus (DI) and oculomotor nerve palsies. Anterior pituitary insufficiency is less commonly seen with pituitary metastasis [1,7-9]. The predilection of metastasis to the posterior lobe of the pituitary gland is likely due to larger contact with the dura and direct supply through the hypophyseal arteries. The anterior pituitary is supplied by the hypophyseal portal system [9]. However, the clinical presentation of pituitary metastasis from RCC tends to differ slightly. Anterior pituitary dysfunction and visual field deficits appear to be common presenting abnormalities, and DI has been described as less common [4,5]. In a literature review of 42 cases, only one patient was found to have an incidental finding of pituitary metastasis, and 10 patients had pituitary metastasis as the initial presentation of RCC [4]. The finding of panhypopituitarism in our case is concordant with the available literature; however, the symptoms of visual and auditory hallucinations have not been reported.

Pituitary metastasis is associated with a poor prognosis [8,10]. Moon et al., in their case series and literature review, reported a one-year mortality of 39% [4]. However, the small case series suggests variable prognosis. Gopan et al. reported only one mortality at the four-year follow-up in their case series of five patients. Improving prognosis could be due to advancements in newer immunotherapies and surgical techniques [5]. The time from diagnosis of RCC to pituitary metastasis has been reported even up to 27 years, with the majority of cases presenting within the first 10 years [4].

Radiological findings of pituitary metastasis could be similar to that of a pituitary adenoma [5,11]. Metastatic lesions to the pituitary usually appear as nodular or irregular thickening. Other possible features include invasion of the surrounding structures, hyperintensity of the optic tracts, vascular flow voids within the tumor, and elimination of characteristic high signal intensity of the posterior lobe [12-14]. Our case had similar imaging findings, which have been mentioned in the literature. Due to its rarity, no specific recommendations exist regarding the best treatment approach for patients with pituitary metastasis from RCC. The intent of treatment is usually palliative. Treatment modalities include surgery, radiation, and chemotherapy [1,4]. No difference in survival outcomes exists between the trans-sphenoidal and transcranial approaches in pituitary metastasis. Surgery may prove beneficial for chiasmal decompression in cases of visual impairment. However, surgery is unlikely to improve the endocrine disturbances [15-17]. Radiotherapy can be used as a primary treatment modality in poor surgical candidates or as an adjuvant therapy after surgery [5]. Newer chemotherapy and immunotherapy agents such as sorafenib and sunitinib have a promising role in the treatment of metastatic RCC [18].

## Conclusions

Metastatic RCC to the pituitary is very rare and most are asymptomatic leading to a delay in diagnosis. Treatment of pituitary metastases is not standardized and should be tailored to patients’ clinical conditions, histology, and the presence of extrapituitary metastases. We need retrospective analysis, meta-analysis, or expert consensus to formulate guidelines for the management of pituitary metastases.
